# Organization of coping strategies and their proximal associations with pain intensity in spinal pain patients

**DOI:** 10.3389/fpain.2026.1824864

**Published:** 2026-08-27

**Authors:** Alemu Sisay Nigru, Federico Maffezzoni, Sergio Benini, Serena Miglio, Matteo Bonetti, Michele Frigerio, Graziella Bragaglio, Chiara D’Adda, Riccardo Leonardi

**Affiliations:** 1 Department of Information Engineering, University of Brescia, Brescia, Italy; 2 X-Ray Service s.r.l., Brescia, Italy; 3 Poliambulatorio Oberdan, Brescia, Italy

**Keywords:** cluster analysis, coping strategies, musculoskeletal pain, pain intensity, pain management, psychological coping, spinal pain

## Abstract

**Background:**

Although coping strategies are well-established psychological correlates of chronic pain, their relative importance and combined organization in relation to pain intensity remain unclear. Clarifying whether coping strategies act as independent correlates or as an organized system is essential for refining psychosocial assessment and intervention.

**Methods:**

In this cross-sectional study, 142 adults with spine-related pain in Brescia, Italy completed the Cope-NVI questionnaire, assessing five coping dimensions. A multi-method analytical framework was applied: multiple regression identified independent associations with pain intensity, bootstrap mediation analyses examined indirect associations among coping strategies, and k-means clustering identified person-centered coping profiles. Sensitivity analyses additionally adjusted for demographic and clinical characteristics.

**Results:**

Multiple regression identified Avoidance and Positive Attitude as the only coping dimensions independently associated with pain intensity. These associations remained significant after adjustment for age, sex, pain duration, and pain location. Bootstrap mediation analyses indicated that associations of Social Support and Problem Orientation with pain intensity were largely accounted for by indirect associations through Avoidance and Positive Attitude. Cluster analysis identified three clinically interpretable coping profiles: Adaptive Copers (low Avoidance, high Positive Attitude; lowest pain), Moderate Copers, and Maladaptive Copers (high Avoidance, low Positive Attitude; highest pain). Cluster differences were observed primarily in coping characteristics and pain intensity rather than demographic or clinical variables.

**Conclusions:**

Coping strategies in spine-related pain appear to be hierarchically organized in relation to pain intensity, with Avoidance and Positive Attitude showing the strongest independent associations. These findings provide a framework for understanding how coping dimensions co-occur within individuals and may help inform psychosocial assessment and person-centered intervention strategies. Given the cross-sectional design, the observed mediation patterns should be interpreted as statistical associations rather than causal mechanisms.

**Significance:**

This study clarifies how coping strategies jointly relate to pain intensity in individuals with spine-related pain. Using integrated regression, mediation, and cluster analyses, it identifies Avoidance and Positive Attitude as the coping dimensions most strongly associated with pain severity and demonstrates that coping strategies organize into clinically interpretable profiles. The findings support psychosocial assessment approaches that prioritize maladaptive avoidance and adaptive cognitive coping processes.

## 1 Introduction

Chronic spinal pain is a quintessential biopsychosocial condition, where psychological factors significantly influence the trajectory of a symptom often initiated by structural pathology (1, 2). While coping, the cognitive and behavioral efforts to manage internal or external demands appraised as taxing, is a well-established psychological determinant, its research faces a pivotal challenge. Decades of study have robustly categorized strategies as broadly adaptive (e.g., acceptance, problem-solving) or maladaptive (e.g., avoidance, catastrophizing) in relation to pain outcomes (3–8). However, this variable-centered literature often treats strategies in isolation, potentially overlooking how they *co-organize within individuals* to shape the pain experience. Consequently, a critical translational gap persists: we lack a granular understanding of which coping dimensions are most proximally tied to pain intensity in clinical samples, and how other, potentially less-direct strategies cluster around these core coping dimentions to form functional or dysfunctional coping profiles. Despite this extensive literature, clinicians still lack empirically grounded guidance on which coping dimensions should be prioritized when pain severity is the primary clinical concern, particularly in spine-related pain where symptom–structure discordance is common.

The prevailing biopsychosocial model necessitates moving beyond simple bivariate relationships to examine the *architecture* of coping (9, 10). Within transactional models of coping, adaptive and maladaptive coping responses are not assumed to contribute equally to pain outcomes. Cognitive appraisal processes, including optimistic reframing and resilience-oriented coping (captured by the Positive Attitude dimension of the CopeNVI), may function as proximal adaptive correlates of adjustment, whereas avoidance-related responses occupy a central role in fear-avoidance models of chronic pain. In contrast, dimensions such as Social Support and Problem Orientation may exert their influence more indirectly by shaping these cognitive and behavioral responses. This perspective raises important questions regarding how coping dimensions are organized and whether some dimensions show stronger associations with pain intensity than others. Answering such questions, consistent with transactional and fear-avoidance models of pain which posit that coping processes influence outcomes through both direct and indirect cognitive-affective mechanisms, requires analytic approaches that can disentangle proximal from distal influences and identify naturally occurring constellations of strategies. Variable-centered methods like multiple regression and mediation are essential to test hierarchical models of influence, while person-centered methods like cluster analysis can reveal whether theoretically coherent subgroups exist in the population, validating and extending findings from the variable-centered level (11–13). A multi-method approach that integrates these levels of analysis offers a pathway to a more nuanced, clinically actionable understanding than either provides alone.

This study therefore aims to address this conceptual and methodological gap in a sample of patients with chronic spinal pain. We employ a coordinated analytic strategy designed to answer three interlocking research questions (RQs):

1. RQ1 (Proximal Correlates): Controlling for other strategies, which specific coping dimensions (positive attitude, problem-solving, social support, avoidance, or transcendent) show the strongest unique association with pain intensity?
2. RQ2 (Organizing Associations): Are the associations between more distal coping strategies (e.g., social support) and pain intensity statistically accounted for by coping dimensions more directly associated with pain intensity (e.g., Positive Attitude or Avoidance)?
3. RQ3 (Natural Co-occurrence): Do individuals naturally cluster into subgroups based on their coping patterns, and do these empirically derived profiles differ meaningfully in reported pain intensity?

While specific interaction effects were explored conservatively, these RQs were guided by theoretical expectations regarding additive vs. hierarchical relationships among coping dimensions. By testing this structured set of questions, we seek to transition from confirming *that* certain coping strategies matter to clarifying *how* they are organized and which are most centrally linked to pain severity. Although pain intensity represents only one dimension of the chronic pain experience, and does not fully capture disability, emotional distress, or quality of life, it remains a central factor influencing healthcare seeking, treatment escalation, and patient distress in spine populations. Consquently, pain intensity was selected as a clinically salient anchor outcome for examining coping-related associations. By identifying the coping dimensions most strongly associated with pain intensity and their natural co-occurrence, this study provides a data-driven framework for personalizing psychological components within multidisciplinary care. For instance, profiles high in maladaptive avoidance may benefit from targeted treatments addressing emotion regulation (14), whereas adaptive clusters could be leveraged to enhance engagement in broader, skills-based programs (15). This reframing has practical implications for efficiently informing the focus of psychological interventions within multidisciplinary spine care.

## 2 Methods

### 2.1 Study design and setting

A cross-sectional observational study was conducted to examine the associations between coping strategies and pain intensity in a clinical sample of patients with spine-related pain. Data were collected at x-ray Service s.r.l., a multidisciplinary outpatient clinic in Brescia, Italy, between August 2024 and September 2025. The study was designed to characterize both independent and combined associations among coping dimensions and pain outcomes within a real-world clinical setting, consistent with a biopsychosocial framework.

### 2.2 Ethical considerations

The study protocol was reviewed and approved by the Ethics Committee of the University of Brescia in accordance with national regulations and institutional requirements (Protocol No. 2024-UNBSCLE-0229777), prior to study commencement. Written informed consent was obtained from all participants before enrollment, in line with the principles of the Declaration of Helsinki. All procedures complied with the European General Data Protection Regulation (GDPR). Patient data were pseudonymized at the clinical site before analysis, and only de-identified datasets were accessed by the research team.

### 2.3 Participants

A clinical sample of 142 adults with spine-related pain was recruited consecutively from the outpatient clinic during the study period. Predefined inclusion and exclusion criteria were applied uniformly to all patients assessed for eligibility. Inclusion criteria required participants to: (1) be aged 18 years or older; (2) have a clinical diagnosis of spine-related pain (lumbar, cervical, or thoracic); and (3) be able to complete self-report questionnaires independently. Exclusion criteria included severe neurological disorders (e.g., neurodegenerative diseases, major neurological deficits, or conditions substantially impairing cognitive or functional capacity), acute psychiatric crises, or inability to understand the study materials. Participation was voluntary and required written informed consent; consequently, not all eligible individuals were enrolled. Detailed demographic and clinical characteristics of the final sample are summarized in Table 1. All data were collected by trained clinic staff using standardized administration procedures.

**Table 1:** Demographic and clinical characteristics of the study sample (*N* = 142).

Characteristic	Total Sample (*N* = 142)
Age, years	
Mean (SD)	51.80 (12.3)
Median (Range)	54.00 (19–87)
Gender, *n* (%)	
Male	85 (59.9%)
Female	57 (40.1%)
Pain Duration, *n* (%)	
>12 weeks (Chronic)	107 (75.4%)
4–12 weeks (Subacute)	24 (16.9%)
<4 weeks (Acute)	11 (7.7%)
Primary Pain Location, *n* (%)	
Lumbar	114 (80.3%)
Sacro-coccygeal	67 (47.2%)
Cervical	34 (23.9%)

Participants could report multiple locations.

### 2.4. Measures

#### 2.4.1 Pain intensity

Pain intensity was assessed using the Numerical Rating Scale (NRS) (16), which measures average pain experienced over the previous 24 h on a scale from 0 (“no pain”) to 10 (“worst imaginable pain”). The NRS is widely used in both clinical practice and pain research and demonstrates strong reliability and construct validity.

#### 2.4.2 Coping strategies

Coping strategies were assessed using the CopeNVI questionnaire (17), the validated Italian short form of the COPE inventory (18). The CopeNVI consists of 25 items organized into five subscales: Social Support (seeking and receiving help from others), Avoidance (withdrawal or denial in response to pain), Positive Attitude (optimistic cognitive reframing and psychological resilience), Problem Orientation (proactive, solution-focused behaviors), and Transcendent Orientation (use of spiritual or meaning-making strategies to handle stress). Responses are rated on a four-point Likert scale ranging from 1 (“I usually don't do it”) to 4 (“I almost always do it”), with higher scores indicating greater use of the corresponding coping strategy.

The CopeNVI has demonstrated good psychometric properties in Italian samples, and COPE-based instruments are widely used in chronic pain research (19–21). In the present sample, all subscales demonstrated good to excellent internal consistency (Cronbach's *α* > 0.80). Table 1 reports descriptive statistics for all coping subscales and pain intensity scores. The complete list of items for the Cope-NVI-25 and Pain NRS is provided in Supplementary File S1.

### 2.5 Statistical analysis

All statistical analyses were conducted using Python (version 3.11.5) with standard scientific libraries (pandas, numpy, scipy, statsmodels). Analyses were planned *a priori* to address the study's research questions by progressively examining independent associations, indirect statistical associations, and person-centered coping profiles.

A sensitivity power analysis was conducted using G*Power (22) to assess whether the available sample size (*N* = 142) provided adequate power for the primary regression analyses. Assuming *α* = .05, power = .80, and all five CopeNVI predictors, the study was sufficiently powered to detect a minimum effect size of f^2^ = 0.087 (equivalent to R^2^ = 0.080). According to Cohen's (1988) conventions, this corresponds to a small effect size.

Descriptive statistics were first computed to summarize demographic characteristics, pain intensity, and coping strategy distributions. Distributional properties of pain intensity were evaluated using visual (histograms, Q–Q plots, box plots) and residual diagnostics to support the use of parametric methods.

Bivariate associations between pain intensity and each coping subscale were examined using both Pearson's and Spearman's correlation coefficients to assess linear relationships while accounting for potential non-normality (23). These analyses provided an initial overview of associations without adjustment for shared variance.

Single-predictor linear regression models were then estimated to quantify the variance in pain intensity explained by each coping strategy independently. Subsequently, a multiple linear regression model including all five coping subscales was specified to estimate their unique associations with pain intensity while controlling for intercorrelations among strategies. To assess the robustness of these associations, a sensitivity analysis was performed using an adjusted model that additionally included demographic and clinical covariates (age, sex, pain duration, and pain location). Model fit was evaluated using R^2^, adjusted R^2^, and overall *F* statistics. Variance inflation factors (VIFs) were examined to assess multicollinearity.

An interaction analysis tested whether the association between Avoidance and pain intensity varied as a function of Positive Attitude, examining whether adaptive and maladaptive coping dimensions operated additively or showed evidence of moderation. Predictors were mean-centered prior to interaction testing to reduce multicollinearity.

Mediation analyses were conducted to examine whether the associations of Social Support and Problem Orientation with pain intensity were statistically accounted for by coping dimensions more associated with pain intensity. Indirect effects were estimated using non-parametric bootstrap resampling (5,000 samples) and 95% bootstrap confidence intervals were calculated. Indirect effects were considered statistically significant when confidence intervals did not include zero. Consistent with recommendations for cross-sectional mediation analyses, results were interpreted as statistical decompositions of associations rather than evidence of causal mechanisms.

Finally, a person-centered cluster analysis was performed to identify naturally occurring coping profiles. Prior to clustering, all coping dimensions were standardized using z-score normalization (*StandardScaler*) to ensure equal contribution to distance calculations. Cluster solutions ranging from *k* = 2 to *k* = 10 were evaluated using the elbow method based on within-cluster sum of squares (24), silhouette coefficients, Davies–Bouldin indices, and bootstrap stability assessment (Jaccard similarity). Initial analyses included all five CopeNVI subscales; however, because Transcendent Orientation showed weak and non-significant associations with pain intensity and reduced overall cluster quality, clustering was repeated using Social Support, Avoidance, Positive Attitude, and Problem Orientation. Although the two-cluster solution yielded the highest silhouette coefficient (0.421 vs. 0.332 for three clusters), the three-cluster solution demonstrated high stability (Jaccard = 0.887), balanced cluster sizes, and preserved a clinically meaningful intermediate coping profile. Therefore, for this exploratory study, the three-cluster solution was retained as the primary person-centered representation of coping patterns. Demographic and clinical characteristics were compared across clusters to assess potential differences in age, sex, pain duration, and pain location. Statistical significance was set at *p* < 0.05 (two-tailed)**.** The Cope-NVI-25 demonstrated high internal consistency in our sample (Cronbach's *α* ≥ .80), which is consistent with its established psychometric properties (17) and previous clinical applications (25).

## 3 Results

The Results section is structured around the three predefined research questions (RQs). First, we identify which coping dimensions show the strongest independent associations with pain intensity (RQ1). Second, we examine whether coping strategies that lose significance in multivariable models are statistically accounted for by coping dimensions more strongly associated with pain intensity (RQ2). Finally, we adopt a person-centered approach to determine whether these associations are reflected in naturally occurring coping profiles with distinct pain levels (RQ3).

### 3.1 Descriptive statistics

The analyzed sample comprised 142 adults with chronic spinal pain. The mean age was 51.8 years (SD = 12.3), 59.9% were male, and most participants reported chronic pain duration (>12 weeks; 75.4%). Lumbar pain was the most frequently reported location (80.3%). Participants reported a mean pain intensity of 5.89 (SD = 2.77) on a 0–10 Numerical Rating Scale, spanning the full range. Descriptive statistics for all Cope-NVI subscales are reported in Table 2, with a graphical summary provided in Supplementary Figure S1.

**Table 2:** Means and standard deviations for all CopeNVI subscales and pain NRS.

Variable	Mean (SD)	N
Social Support	2.47 (0.79)	142
Avoidance	1.74 (0.92)	142
Positive Attitude	2.72 (1.04)	142
Problem Orientation	2.88 (0.81)	142
Transcendent	1.64 (0.78)	142
Pain NRS	5.89 (2.77)	142

### 3.2 RQ 1: independent associations of coping dimensions with pain intensity

To address RQ1 and identify coping dimensions most strongly associated with pain intensity, a multiple linear regression model was estimated including all five Cope-NVI subscales simultaneously. The model was statistically significant and explained 41.4% of the variance in pain intensity [R^2^ = 0.414, adjusted R^2^ = 0.392; F(5, 136) = 19.18, *p* < 0.001]. Sensitivity analysis indicated that the available sample size was sufficient to detect small effects (minimum detectable f^2^ = 0.094), while the observed model demonstrated a substantially larger effect size (R^2^ = 0.414; f^2^ = 0.707).

As shown in Table 3, Avoidance emerged as the strongest independent correlate of higher pain intensity [*β* = 0.474; B = 1.428; SE = 0.197; *p* < 0.001; 95% CI (1.041, 1.815)]), whereas Positive Attitude showed a significant inverse association with pain [*β* = −0.293; B = −0.781; SE = 0.174; *p* < 0.001; 95% CI (−1.122, −0.439)]. Social Support, Problem Orientation, and Transcendent Orientation were not independently associated with pain intensity after accounting for shared variance among coping dimensions. To assess the robustness of these findings, an adjusted regression model was estimated including age, sex, pain duration, and pain location (*N* = 130 complete cases; Table 4). The adjusted model remained statistically significant [R^2^ = 0.394, adjusted R^2^ = 0.331; F(12,117) = 6.33, *p* < .001]. Avoidance (*β* = 0.413, *p* < .001) and Positive Attitude (*β* = −0.260, *p* = .008) remained the only coping dimensions independently associated with pain intensity, whereas demographic and clinical covariates were not significant independent correlates.

**Table 3:** Multiple regression analysis: All CopeNVI subscales predicting pain intensity (NRS).

**Predictor**	**B (SE)**	***β***	**p-value**	**95% CI**
Social Support	0.244 (0.230)	0.069	0.100	[−0.207, 0.696]
Avoidance	1.428 (0.197)	0.474	*<0*.*001*	[1.041, 1.815]
Positive Attitude	−0.781 (0.174)	−0.293	*<0*.*001*	[−1.122, −0.439]
Problem Orientation	0.187 (0.224)	0.055	0.100	[−0.251, 0.626]
Transcendent	−0.187 (0.232)	−0.053	0.100	[−0.642, 0.269]

Values in italics indicate the statistical significance of the model parameters. Model R² = 0.414, Adjusted R² = 0.392, F(5, 136) = 19.182, *p* < 0.001. A sensitivity power analysis indicated that the available sample (*N* = 142) was sufficient to detect small effects (minimum detectable f² = 0.094; R² = 0.086).

**Table 4:** Adjusted multiple regression model predicting pain intensity (pain NRS) including coping dimensions and demographic/clinical covariates (*N* = 130).

**Predictor**	**B (SE)**	**β**	**p**	**95% CI**
Social Support	0.162 (0.338)	0.046	.633	[−0.508, 0.832]
Avoidance	1.323 (0.311)	0.413	<.001	[0.707, 1.938]
Positive Attitude	−0.697 (0.259)	−0.260	.008	[−1.210, −0.185]
Problem Orientation	0.062 (0.283)	0.018	.826	[−0.498, 0.623]
Transcendent	−0.243 (0.258)	−0.071	.347	[−0.754, 0.267]
Age	0.004 (0.016)	0.017	.818	[−0.029, 0.036]
Female	0.707 (0.430)	0.128	.103	[−0.145, 1.559]
Pain duration (Subacute)	−0.955 (0.546)	−0.133	.083	[−2.035, 0.126]
Pain duration (Acute)	0.217 (0.754)	0.022	.774	[−1.276, 1.711]
Lumbar pain	−0.032 (0.666)	−0.005	.962	[−1.352, 1.288]
Cervical pain	0.763 (0.593)	0.120	.201	[−0.411, 1.937]
Sacro-coccygeal pain	0.395 (0.422)	0.073	.352	[−0.442, 1.231]

Model statistics: R^2^ = 0.394; Adjusted R^2^ = 0.331; F(12,117) = 6.33; *p* < .001—Statistically significant.

For completeness, bivariate correlations between pain intensity and all CopeNVI subscales are reported in Supplementary Table S1, and their scatterplot distributions with regression lines are visualized in Supplementary Figure S2.

Interaction analysis demonstrated no significant Avoidance × Positive Attitude interaction effect (*β* = 0.025, *p* = .831). Both coping dimensions remained independently associated with pain intensity, supporting additive rather than multiplicative associations between adaptive and maladaptive coping processes.

### 3.3 RQ 2: indirect associations among coping strategies

Mediation analyses were conducted to examine whether the associations of Social Support and Problem Orientation with pain intensity were statistically accounted for by Avoidance and Positive Attitude, the two coping dimensions identified as independent correlates in RQ1.

#### 3.3.1 Mediation involving social support

The total association between Social Support and pain intensity was significant (c = −1.138, *p* < .001). As shown in Table 5, this association was largely accounted for by indirect associations through both Positive Attitude and Avoidance. Specifically, higher Social Support was associated with higher Positive Attitude, which in turn was associated with lower pain intensity, yielding a significant indirect effect [indirect effect = −0.969, 95% CI (−1.378, −0.620)]. This pathway accounted for approximately 85% of the total association between Social Support and pain.

**Table 5:** Mediation analysis: avoidance and positive attitude as mediators between other coping strategies and pain intensity.

Predictor → Mediator → Outcome	a (SE)	b (SE)	Indirect Effect (a × b)	95% CI	% Mediated
Problem Orientation → Positive Attitude → Pain	0.495 (0.100)	−1.441 (0.189)	−0.713 (0.172)	[−1.026, −0.440]	115.9%^***^
Problem Orientation → Avoidance → Pain	−0.375 (0.091)	1.813 (0.204)	−0.680 (0.181)	[−1.037, −0.333]	109.3%^***^
Social Support → Avoidance → Pain	−0.630 (0.083)	1.813 (0.204)	−1.142 (0.198)	[−1.574, −0.770]	100.4%^***^
Social Support → Positive Attitude → Pain	0.706 (0.094)	−1.441 (0.189)	−1.017 (0.191)	[−1.376, −0.620]	85.1%^***^

Indirect effects *e*stimated using 5,000 bootstrap resamples. Confidence intervals not crossing zero were considered significant).

*** *p* < 0.001.

In parallel, higher Social Support was associated with lower Avoidance, which was strongly associated with higher pain intensity, producing a significant indirect effect [indirect effect = −1.143, 95% CI (−1.574, −0.770)]. This indirect association accounted for virtually all of the total association, rendering the direct effect of Social Support on pain non-significant (c′ = 0.021, *p* = .916). Together, these findings indicate that the association between Social Support and pain intensity was largely statistically accounted for by coping dimensions more strongly associated with pain intensity.

#### 3.3.2 Mediation involving problem orientation

The total association between Problem Orientation and pain intensity was not statistically significant (c = −0.628, *p* = .056). However, consistent with contemporary mediation frameworks, significant indirect effects were observed despite the absence of a total association, indicating statistical suppression.

Problem Orientation showed a strong positive association with Positive Attitude, which was in turn associated with lower pain intensity, yielding a significant indirect effect [indirect effect = −0.728, 95% CI (−1.026, −0.440)]. This indirect association exceeded the magnitude of the total association, indicating suppression of a small opposing direct association.

Similarly, higher Problem Orientation was associated with lower Avoidance, producing an additional significant indirect association with pain intensity [indirect effect = −0.686, 95% CI (−1.037, −0.333)]. The direct effect of Problem Orientation on pain remained non-significant (c′ = 1.248, *p* = .058).

#### 3.3.3 Interpretation of indirect effects

Across models involving both Social Support and Problem Orientation, indirect associations through Avoidance and Positive Attitude were consistently observed. In several models, indirect effects exceeded the magnitude of the total association, indicating statistical suppression, whereby indirect associations accounted for relationships that were not evident in the total association estimate. Given the cross-sectional design, these findings should be interpreted as statistical decompositions of associations rather than evidence of causal mechanisms. Results for all other tested mediation models are provided in Supplementary Table S3.

### 3.4 RQ 3: person-centered coping profiles

Following preliminary analyses, Transcendent Orientation was excluded from the clustering procedure because it showed weak and non-significant associations with pain intensity and reduced overall cluster quality. K-means clustering was therefore performed using four standardized Cope-NVI dimensions: Social Support, Avoidance, Positive Attitude, and Problem Orientation.

Cluster solutions ranging from k = 2 to k = 10 were evaluated using the elbow method, silhouette coefficients, Davies–Bouldin indices, and bootstrap stability assessment (Table 6; Supplementary Figure S4). Although the two-cluster solution yielded the highest silhouette coefficient (0.421), the three-cluster solution demonstrated high stability (bootstrap Jaccard = 0.887), balanced cluster sizes, and preserved a clinically meaningful intermediate coping profile. Therefore, for this exploratory study, the three-cluster solution was retained for primary interpretation.

**Table 6:** Cluster validation metrics.

Solution	Silhouette	Davies–Bouldin	Bootstrap Jaccard	Cluster Sizes
*k* = 2	0.421	0.991	0.891	42, 100
*k* = 3	0.332	1.148	0.887	72, 45, 25

**Figure 1:**
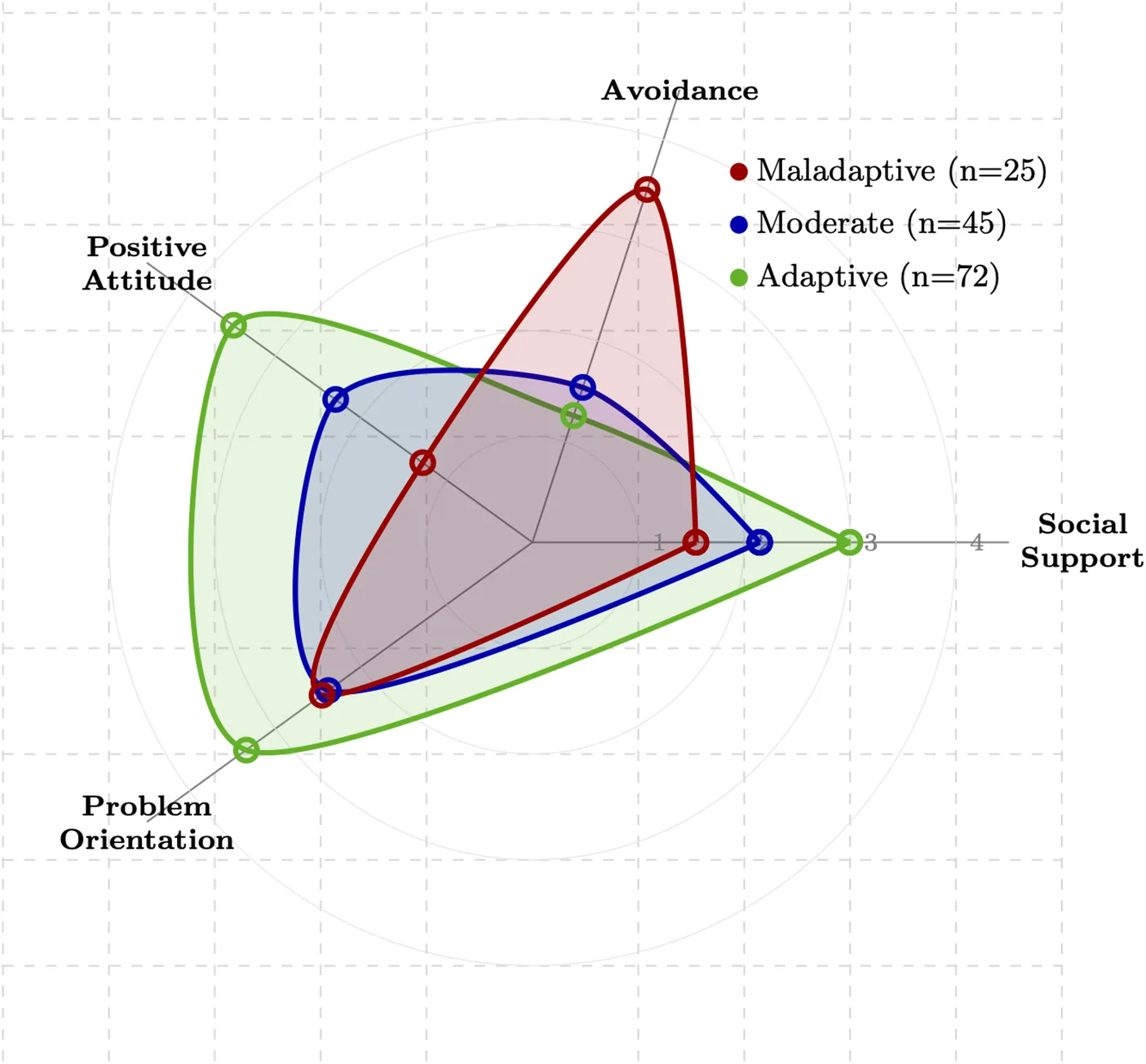
Radar plot showing the three coping profiles identified using k-means clustering on four CopeNVI subscales (social support, avoidance, positive attitude, and problem orientation). Adaptive Copers (*n* = 72, 50.7%) demonstrated high level of adaptive strategies (Positive Attitude µ = 3.49, Problem Orientation µ = 3.34, Social Support µ = 3.00) and low Avoidance (µ = 1.26), and reported the lower pain intensity (µ = 4.79). Maladaptive Copers (*n* = 25, 17.6%) exhibited elevated Avoidance (µ = 3.50) together with low adaptive strategies (Positive Attitude µ = 1.28, Social Support µ = 1.54), with highest pain intensity (µ = 9.00). Moderate Copers (*n* = 45, 31.7%) showed intermediate levels across all coping dimensions, with moderate pain intensity (µ = 5.91).

The resulting clusters were labeled according to their mean pain intensity as Adaptive Copers (*n* = 72), Moderate Copers (*n* = 45), and Maladaptive Copers (*n* = 25). As illustrated in Figure 1, Adaptive Copers demonstrated high Positive Attitude, Problem Orientation, and Social Support with low Avoidance, whereas Maladaptive Copers showed markedly elevated Avoidance alongside lower adaptive coping dimensions. Moderate Copers exhibited intermediate values across most coping dimensions.

As shown in Supplementary Table S2, significant differences were observed across clusters for all four coping dimensions (all *p* < .001). Adaptive Copers demonstrated the highest levels of Positive Attitude (M = 3.49, SD = 0.65), Problem Orientation (M = 3.34, SD = 0.57), and Social Support (M = 3.00, SD = 0.57), together with the lowest Avoidance scores (M = 1.26, SD = 0.33). In contrast, Maladaptive Copers exhibited markedly elevated Avoidance (M = 3.50, SD = 0.37), low Positive Attitude (M = 1.28, SD = 0.23), and low Social Support (M = 1.54, SD = 0.42). Moderate Copers displayed intermediate values across all coping dimensions.

Pain intensity differed significantly across profiles (Kruskal–Wallis *p* < .001). Adaptive Copers reported the lowest pain intensity (M = 4.79, SD = 2.90), Moderate Copers reported intermediate pain intensity (M = 5.91, SD = 2.90), and Maladaptive Copers reported the highest pain intensity (M = 9.00, SD = 0.71). Age, sex, pain duration, and pain location did not differ significantly across clusters, suggesting that the identified profiles primarily reflected coping characteristics rather than demographic or clinical differences.

### 3.5 Supplementary analysis: model diagnostics

Diagnostic assessments confirmed that the assumptions for the primary regression analyses were adequately met. Histograms and Q-Q plot indicated that pain intensity scores and model residuals approximated normal distributions. Variance inflation factors were low across all predictors (all VIFs < 2), indicating minimal multicollinearity. Residual plots from both the primary and adjusted multiple regression models showed residuals symmetrically distributed around zero with approximately constant variance, supporting assumptions of normality, linearity, and homoscedasticity (Supplementary Figure S3).

## 4 Discussion

This study advances the understanding of coping in chronic spinal pain by demonstrating that coping strategies are not independent correlates of pain intensity, but are hierarchically organized. By integrating regression, mediation, and person-centered clustering approaches, Avoidance and Positive Attitude emerged as the coping dimensions most strongly and independently associated with pain intensity, while other coping strategies, such as Social Support and Problem Orientation, were statistically associated with pain intensity primarily through indirect associations involving these core coping dimensions. This organizational perspective moves beyond cataloguing adaptive and maladaptive strategies and clarifies how coping processes are structured in relation to pain intensity.

Variable-centered analyses revealed a clear dual-pathway pattern. Avoidance and Positive Attitude were the only coping dimensions that retained independent associations with pain intensity when all strategies were considered simultaneously, together accounting for over 40% of the variance. This suggests that maladaptive behavioral disengagement and adaptive cognitive appraisal represent distinct, proximal associations. The centrality of Avoidance aligns with extensive fear-avoidance literature (20) and is powerfully underscored by contemporary research showing that daily passive coping, a close analogue, is linked to worse daily functional and psychosocial outcomes (7). Conversely, the unique role of Positive Attitude resonates with evidence that adaptive cognitive strategies like reappraisal can buffer the impact of pain on daily functioning (12).

An additional finding was the limited role of Transcendent Orientation. Unlike Avoidance and Positive Attitude, Transcendent Orientation did not retain an independent association with pain intensity in multivariable analyses and contributed minimally to the organization of coping profiles. Participants generally reported relatively low levels of transcendent coping, suggesting that spiritual or meaning-based coping strategies were not a prominent feature of coping behavior within this cohort. Importantly, this finding should not be interpreted as evidence that transcendent coping lacks relevance in chronic pain. Previous work has suggested that spiritual and meaning-oriented coping may contribute to resilience, psychological adjustment, and adaptation to chronic illness, although its role appears highly dependent on cultural, contextual, and individual factors (26–28). Accordingly, the limited contribution observed in the present study may reflect the characteristics of this specific clinical population rather than an absence of potential adaptive value. Consistent with this interpretation, excluding Transcendent Orientation improved cluster quality and stability, indicating that it contributed relatively little to the coping architecture most strongly associated with pain intensity in this sample.

Crucially, mediation analyses demonstrated that the apparent bivariate associations of Social Support and Problem Orientation with pain were almost entirely transmitted through the core associations of Avoidance and Positive Attitude, a pattern consistent with models in which coping dimensions are statistically interrelated and may account for observed associations between psychosocial resources and pain-related outcomes (29–31). This pattern aligns with evidence from other chronic conditions, where specific coping strategies have been shown to mediate the relationship between psychological factors and patient-reported outcomes (32). This pattern of suppression effects helps resolve inconsistencies in prior coping research by showing that some strategies operate primarily as distal or organizing resources rather than coping dimensions more strongly associated with pain intensity (33). For instance, Social Support may confer benefit by reducing avoidance tendencies and fostering positive appraisals, rather than through a direct analgesic effect.

The person-centered cluster analysis provided convergent support for this organizational pattern. The three emergent coping profiles: Adaptive, Moderate, and Maladaptive Copers, were defined principally by their levels of Avoidance and Positive Attitude. This finding empirically validates the dual-pathway model at the patient level and is consistent with longitudinal data showing that patients often exhibit coherent patterns of active or passive coping over time (8). The Maladaptive Coper profile, characterized by high Avoidance and low Positive Attitude, mirrors groups identified in other chronic pain populations who report the highest pain and poorest adjustment (5, 8).

From a clinical perspective, these findings suggest that assessment should prioritize identifying levels of Avoidance and Positive Attitude, as these were the coping dimensions most strongly associated with pain intensity. This supports a stratified care approach where patients with a Maladaptive Coper profile might first require interventions targeting avoidance reduction and cognitive reappraisal (34), while those with adaptive profiles may benefit from strengthening existing strengths. This tailored strategy aligns with the principles of effective multidisciplinary pain programs, which integrate psychological and physical approaches (15, 35). Notably, our findings also imply that promoting Social Support or Problem-Solving in isolation, without addressing the core proximal associations of avoidance and appraisal, may be insufficient to optimize pain-related outcomes if avoidance-related and appraisal-related coping processes are not simultaneously considered. This approach is consistent with evidence supporting broader coping-focused interventions, such as Pain Coping Skills Training, which effectively improves multiple pain-related outcomes (36).

Several limitations warrant consideration, including the cross-sectional design precludes causal inference, and therefore the observed indirect associations should not be interpreted as evidence of causal mechanisms. Second, pain intensity was the primary outcome and represents only one dimension of the chronic pain experience. Measures of disability, emotional distress, catastrophizing, and broader quality-of-life outcomes were not incorporated into the primary analytical framework. Consequently, the findings should be interpreted as relating specifically to pain intensity rather than chronic pain adjustment more broadly. Future longitudinal and interventional research is needed to test whether changes in Avoidance and Positive Attitude mediate improvements in pain outcomes following treatment, and to explore the stability of the identified coping profiles over time as discussed in (8). Reliance on self-report measures and a single cultural context may also limit generalizability.

## 5 Conclusions

Across multiple analytical approaches, Avoidance and Positive Attitude consistently emerged as the coping dimensions most strongly associated with pain intensity, clarifying a hierarchical organization within the coping system in chronic spinal pain. Associations involving other strategies, including Social Support and Problem Orientation, was largely indirect, channeled through these core associations. The identified coping profiles validate this structure at the person level, underscoring the clinical value of integrating variable-centered and person-centered perspectives. Collectively, these findings provide a parsimonious organizational framework for understanding how coping dimensions relate to pain intensity in individuals with chronic spinal pain. By integrating variable-centered and person-centered perspectives, the proposed framework highlights how coping strategies may be systematically organized in relation to pain intensity and identifies potential targets for stratified assessment and intervention, while noting that causal inferences require future longitudinal and interventional study.

## Data Availability

The raw data supporting the conclusions of this article will be made available by the authors, without undue reservation.
