# Supplementary material for: Organization of coping strategies and their proximal associations with pain intensity in spinal pain patients
Source: Front Pain Res (Lausanne). 2026 Aug 27;7:1824864. doi: 10.3389/fpain.2026.1824864 (PMC13558419; doi:10.3389/fpain.2026.1824864)
Supplement: Supplementary file 1 [file Table1.docx]

## **Organization of Coping Strategies and Their Proximal Associations with Pain Intensity in Spinal Pain Patients**

**Supplementary tables**

Supplementary Table S1: Correlation matrix of pain intensity (NRS) with CopeNVI subscales.

| **CopeNVI**  **Subscale** | **Pearson’s r** | **p-value** | **N** |
| --- | --- | --- | --- |
| Social Support | -0.323 | <0.001 | 142 |
| Avoidance | 0.601 | <0.001 | 142 |
| Positive Attitude | -0.541 | <0.001 | 142 |
| Problem Orientation | -0.183 | 0.050 | 142 |
| Transcendent | -0.049 | 0.050 | 142 |

Supplementary Table S2: Cluster Characteristics

| **Variable** | **Adaptive (n=72)** | **Moderate (n=45)** | **Maladaptive (n=25)** | **p** |
| --- | --- | --- | --- | --- |
| Social Support | 3.00 ± 0.57 | 2.15 ± 0.55 | 1.54 ± 0.42 | <0.001 |
| Avoidance | 1.26 ± 0.33 | 1.54 ± 0.50 | 3.50 ± 0.37 | <0.001 |
| Positive Attitude | 3.49 ± 0.65 | 2.30 ± 0.66 | 1.28 ± 0.23 | <0.001 |
| Problem Orientation | 3.34 ± 0.57 | 2.38 ± 0.69 | 2.46 ± 0.86 | <0.001 |
| Pain NRS | 4.79 ± 2.28 | 5.91 ± 2.90 | 9.00 ± 0.71 | <0.001 |

Supplementary Table S3: Additional exploratory mediation analyses among coping dimensions and pain intensity.

| Predictor → Mediator → Outcome | a (SE) | b (SE) | Indirect Effect (a×b) | 95% CI | % Mediated |
| --- | --- | --- | --- | --- | --- |
| Avoidance → Positive Attitude → Pain | -0.719 (0.074) | -1.441 (0.189) | 1.036 (0.173) | [0.716, 1.352] | 57.1%^∗∗^ |
| Positive Attitude → Social Support → Pain | 0.404 (0.054) | -1.139 (0.282) | -0.460 (0.129) | [-0.755, -0.202] | 31.9%^∗∗^ |
| Avoidance → Social Support → Pain | -0.462 (0.061) | -1.139 (0.282) | 0.526 (0.147) | [0.242, 0.842] | 29.0%^∗∗^ |
| Social Support → Problem Orientation → Pain | 0.449 (0.078) | -0.628 (0.284) | -0.282 (0.137) | [-0.593, 0.001] | 24.8%^∗^ |
| Positive Attitude → Problem Orientation → Pain | 0.300 (0.061) | -0.628 (0.284) | -0.188 (0.093) | [-0.432, 0.001] | 13.1%^∗^ |
| *Note: Indirect effects were estimated using nonparametric bootstrap mediation analyses with 5,000 resamples.*  *(^**^ statistically significant, ^*^ statistically not significant).* | | | | | |
